# Single cell meta-analysis of EndMT and EMT state in COVID-19

**DOI:** 10.3389/fimmu.2022.976512

**Published:** 2022-09-29

**Authors:** Lanlan Zhang, Chuang Tang, Min Zhang, Xia Tong, Yingying Xie, Ruitong Yan, Xiangjun Wang, Xin Zhang, Dan Liu, Shasha Li

**Affiliations:** ^1^ Division of Pulmonary Diseases, State Key Laboratory of Biotherapy, And Department of Respiratory and Critical Care Medicine, West China Hospital of Sichuan University, Chengdu, China; ^2^ Department of Gastroenterology, West China (Airport) Hospital, Sichuan University, Chengdu, China; ^3^ Oncology Bussiness Department, Novogene Co., Ltd, Beijing, China; ^4^ Department of Gastroenterology, West China Hospital of Sichuan University, Chengdu, China; ^5^ Department of Nephrology, Seventh Affiliated Hospital Sun Yat-sen University, Shenzhen, China; ^6^ Sichuan University, Chengdu, China; ^7^ First Affiliated Hospital of Sun Yat-Sen University, Guangzhou, China; ^8^ Athinoula A. Martinos Center for Biomedical Imaging, Department of Radiology, Massachusetts General Hospital, Charlestown, MA, United States; ^9^ Harvard Medical School, Boston, MA, United States

**Keywords:** COVID-19, endothelial-mesenchymal transition (EndMT), epithelial-mesenchymal transition (EMT), single cell RNA sequencing, endothelial cells (ECs)

## Abstract

COVID-19 prognoses suggests that a proportion of patients develop fibrosis, but there is no evidence to indicate whether patients have progression of mesenchymal transition (MT) in the lungs. The role of MT during the COVID-19 pandemic remains poorly understood. Using single-cell RNA sequencing, we profiled the transcriptomes of cells from the lungs of healthy individuals (n = 45), COVID-19 patients (n = 58), and idiopathic pulmonary fibrosis (IPF) patients (n = 64) human lungs to map the entire MT change. This analysis enabled us to map all high-resolution matrix-producing cells and identify distinct subpopulations of endothelial cells (ECs) and epithelial cells as the primary cellular sources of MT clusters during COVID-19. For the first time, we have identied early and late subgroups of endothelial mesenchymal transition (EndMT) and epithelial-mesenchymal transition (EMT) using analysis of public databases for single-cell sequencing. We assessed epithelial subgroups by age, smoking status, and gender, and the data suggest that the proportional changes in EMT in COVID-19 are statistically significant. Further enumeration of early and late EMT suggests a correlation between invasive genes and COVID-19. Finally, EndMT is upregulated in COVID-19 patients and enriched for more inflammatory cytokines. Further, by classifying EndMT as early or late stages, we found that early EndMT was positively correlated with entry factors but this was not true for late EndMT. Exploring the MT state of may help to mitigate the fibrosis impact of SARS-CoV-2 infection.

## Introduction

Alveolar epithelial type II cells (AEC2s) produce surfactants and serve as progenitors (1). Type II alveolar epithelium is reduced due to massive necrosis with SARS-COV-2 infection in patients (2). This leads to decreased barrier permeability and accumulation of proteinaceous edema fluid in the alveolar cavity, resulting in hypoxemia and even acute respiratory distress syndrome (ARDS), with a prognosis including fibrosis formation (3). Epithelial-mesenchymal transition (EMT) is the process by which epithelial cells differentiate into mesenchymal (fibroblast-like) cells expressing mesenchymal biomarkers such as α-Smooth Muscle Actin (α-SMA), and N-cadherin (4, 5). Therefore, we speculated that EMT might underlie the mechanism of lung fibrosis observed with ARDS in COVID-19 patients.

Early-stage and late-stage mesenchymal transition (MT) processes are considered the most critical pathogenic mechanisms of fibrosis in diseases like idiopathic pulmonary fibrosis (IPF), pulmonary hypertension, coronary artery disease, and other vascular-related disorders (6). Once endothelial cells (ECs) are damaged and the collagen and matrix are exposed, in addition to activation of the coagulation system, immune cells are recruited, leading to the release of large amounts of inflammatory factors are then released (7). Endothelial-mesenchymal transition (EndMT) occurs when ECs respond to chronic inflammation, transforming themselves into a more aggressive mesenchymal state (8, 9). In the early stages, irreversible vascular damage or EndMT, mainly presents more robust endothelial markers (e.g., vascular endothelial cadherins (VE-cadherins), *CD31* and Tie1/2) and expresses weaker mesenchymal markers (e.g., *N-cadherin*, *fibroblast specific protein-1* or *S100A4*, *fibronectin*, *vimentin*, *SM22-α*, *calponi*n, and *α-smooth muscle actin*) (10, 11). Otherwise, late-stage EndMT presents weaker endothelial markers but with more robust mesenchymal markers.

EMT also has a similar process with the presence of EndMT as well as MT markers in the early stages (12, 13). However, in the late stage, epithelial markers are lost, and fibroblast markers are predominant (12, 13). Therefore, we hypothesized that EMT and EndMT may underlie factors implicated in the prolonged ventilator dependence and high mortality rates observed in hospitalized COVID-19 patients.

Herein, we draft EndMT and EMT by integrated analysis of 167 single-cell and single-nucleus RNA-sequencing (scRNA-seq and snRNA-seq, respectively) samples. We performed the first single-cell RNA sequencing meta-analysis associating COVID-19 with underlying contributor genes for MT. We have also identified specific gene programs enriched in MT-associated genes with fibrosis and highlight other entry factors that are significantly expressed in the lungs, which may play a role in SARS-CoV-2 infection.

## Materials and methods

### Data collection

We obtained scRNA/snRNA sequencing data from Gene Expression Omnibus database (GEO) database (https://www.ncbi.nlm.nih.gov/geo/). The majority of the data used in this manuscript is publicly available from previously published studies: COVID-19:GEO accession GSE171524, GSE171668, GSE149878, GSE161382 and GSE163919 (14–17). IPF: GEO accession GSE136831 and GSE135893 (18, 19). The Bulk RNA data is also available in the GEO database (accession number GSE47460) (20).

### scRNA/snRNA-seq data processing

We analyzed the scRNA and snRNA sequencing data using R(version 4.1.3) and Seurat v 4.0.2 (21). To eliminate low quality/dead cells or empty droplets, any single cells containing less than 200 genes or greater than 5,000 genes, or cells with more than 10% mitochondrial genes were removed. We next removed doublet contamination. As a result. We detected 49,795 genes in a total of 612,151 cells. Next, we performed LogNormalize() to normalize the gene expression data, used the FindVariableFeatures() function to identify genes whose expression was highly variable between cells, and used ScaleData() to scale the data. The •"RunPCA•" function was used for principal component analysis (PCA). Using the default parameters, Harmony package (22) was utilized to combine data and eliminate batch effect, Using the default parameters, Harmony performs de-batching by single patients. Subsequently, the top 20 statistically significant principal components were used in the "•FindNeighbors•" function. Cells were clustered (Cluster resolution = 0.6) by using the •FindClusters• function and visualized with the UMAP method.

### Cell type identification and data visualization

Cell type identification was mostly accomplished with the use of “FindAllMarkers,” classical cell marker genes, R-packages (clustermole, singscore), and cell type annotation using a mixture of known cell marker genes. *EPCAM^+^* epithelial cells, *CLDN5^+^* endothelial cells, *PTPRC^+^* immune cells, and *COL1A2^+^* (mesenchymal cells). Cell clustering was performed using the Seurat “FindNeighbors” and “FindClusters” functions, and the final Seurat objects were created after UMAP downscaling. The pheatmap was created using the R package ggplot2 in Seurat, and umap maps, violin maps, bar maps, heat maps, and point maps were created.

### Enrichment analysis

Enriched gene ontology biological (http://geneontology.org/) processes with a false discovery rate less than 5 percent were identified using the Gene Ontology Resource. KEGG (http://www.kegg.jp) enrichment analyses were carried out with the Fisher’s exact test, and FDR correction for multiple testing was also performed. Gene Set Enrichment Analysis (GSEA) were performed with clusterProfile. The three methods determine whether a set of genes has statistically significant differences between two biological states (23).

### Trajectory analysis

The R package monocle3 v1.0.0 was used to construct single cell trajectory analysis. Cells marked as EndMT/EMT and their subsets information were input and constructed into a monocle object (24). The left side of the heat map, created with the R package monocle2, shows the signaling pathways enriched to the gene set. Each row of the heat map (ranging from red to blue) represents a gene; each column represents a proposed time point; and the color represents the average expression value of the gene at the current time point (from high to low expression, respectively) (25).

### Bulk RNA data

Univariate analysis was performed by using the Pearson’s correlation coefficient of gene expression data and diffusing capacity of the lung for carbon monoxide (DLCO). The data is available in the Gene Expression Omnibus database (accession number GSE47460).

### Statistical analysis of functional data

The R language or the GraphPad Prism software, version 9 (San Diego, California USA, www.graphpad.com) were used to perform all computations and analyses. T-tests were used for comparisons between groups, and one-way ANOVA was used for comparisons between multiple groups. A statistically significant difference was defined as a p value <0.05. The Wilcoxon rank sum statistical test was used to examine differentially expressed genes in each cluster. The meta package was used to perform the meta-analysis in R.

## Results

### Single-cell transcriptome sequencing reveals the presence of fibrosis-associated cell subpopulations

**Figure 1:**
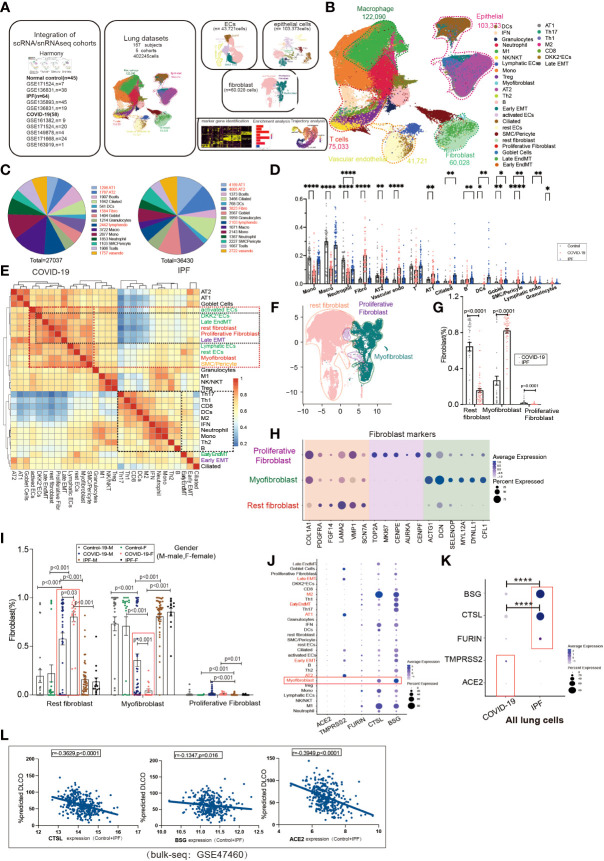
Subpopulation correlation analysis suggested a strong correlation between MT subpopulation and fibroblast subpopulation. **(A)** Scheme of this study. **(B)** Demonstration of HC, COVID-19, and IPF subgroups using UMAP, including Endothelial (vascular endothelial: rest ECs, EndMT, DKK2+ ECs, activated ECs; Lymphatic ECs); Epithelial (AT2, AT1, Goblet Cell, EMT, Ciliated); Fibroblast (Proliferative Fibroblast, Myofibroblast, Rest fibroblast, Late EndMT, Late EMT); SMC/Pericyte; T cells (Th1, Th17, Th2, Treg, NK/NKT, CD8, IFN); Macrophage (M2, M1); Granulocytes; DCs; Mono; Neutrophil. And the proportion of cell subpopulations in HC, COVID-19, IPF. Marker genes: Endothelial cells:PECAM1, VWF, CLDN5; Macrophages: MARCO, MSR1, MRC1;T cells:CD3E, CD3D, GZMH; Granulocytes:MS4A2, CPA3, TPSAB1; B cells:MS4A1, BANK1, CD79A;Monocytes:CD14, FCN1; Neutrophil:S100A8, S100A9; Epithelial cells:EPCAM; Fibroblast:COL1A1, PDGFRA, ELN;SMC:SCGB1A1, RPL26). **(C)** Pie charts show the number of differentially expressed genes per cell type in COVID-19/IPF compared to controls. The DEGs of COVID-19 were mainly concentrated in macrophages and monocytes, and the DEGs of IPF were mainly concentrated in epithelium. **(D)** The proportion of cell subpopulations in HC, COVID-19, IPF. **(E)** The heat-map shows the correlation analysis for each cell subpopulation. The correlation between late-stage EMT, late-stage EndMT and fibroblast subpopulation was higher. **(F)** UMAP suggests a subpopulation distribution of fibroblasts, including Myofibroblas,Proliferative fibroblas and Rest fibroblast. **(G)** Ratio of fibroblast subpopulations in patients with COVID-19, IPF, showing myofibroblast ratio in IPF is higher than that in COVID-19, but Rest fibroblast ratio in IPF is lower than that in COVID-19. **(H)** Markers of fibroblast subpopulations, including Myofibroblast (ACTG1, DCN, SELENOP, MYL12A, DYNLL1, CFL1), Proliferative fibroblast (TOP2A, MKI67, CENPE, AUPKA, CENPF) and Rest fibroblast (COL1A1, PDGFRA, FGF14, LAMA2, VMP1, SCN7A). **(I)** The proportion of fibroblast subpopulations in COVID-19, IPF by female and male, showing Myofibroblast ratio in females is lower than that in males with COVID-19. **(J)** The DOT plot shows the expression of entry factors in subgroups of control, COVID-19, IPF. Subgroups including Rest fibroblast, Myofibroblast, Proliferative Fibroblast, Late EndMT, Late EMT, SMC/Pericyte; rest ECs, DKK2^+^ ECs, activated ECs, Lymphatic ECs, EndMT-early; AT1, AT2, Ciliated, Early EMT, Goblet Cell; Granulocytes; B, Th2, Th1, Th17, Treg, CD8, Mono, M1, M2, Neutrophil, DCs, NK/NKT. **(K)** The DOT plot shows the expression of entry factors in COVID-19, IPF. **(L)** Correlation analysis of entry factors (CTSL, BSG, ACE2) and lung function test (%predicted DLCO) showed that lung function was negatively correlated with the normalized bulk RNA-seq gene expression of the entry factors. *P < 0.05, **P < 0.01,**** P< 0.0001.

To investigate the contribution of EndMT in SARS-CoV-2 infection in the lungs, we analyzed existing scRNA/snRNA-seq datasets to assess which clusters express EndMT markers. In a previously unpublished dataset consisting of COVID-19 and IPF lung tissue (*n*=167), we recovered at least 14 distinct major cell types, including macrophages, monocytes, neutrophils, T cells, B cells, DCs, granulocytes, endothelial cells, fibroblasts, SMC/pericytes, goblet cells, alveolar type 1 epithelial cells (AT1), alveolar type 2 epithelial cells(AT2), ciliated cell populations, and proportional analysis of subpopulations, of whole lung tissue (**Figures 1A, B**). After analyzing their differentially expressed genes (DEGs), compared to controls, we found that the DEGs in COVID-19 patients were mainly concentrated in macrophages and monocytes, and the DEGs in IPF patients were mainly concentrated in the epithelium (**Figure 1C**). Further, we analyzed the ratio of cell subpopulations and found that the ratio of ECs and epithelial was upregulated in COVID-19 patients, while higher than in pulmonary fibrosis (**Figure 1D**). Using correlation analysis, we found that lymphatic ECs, SMC/pericytes, myofibroblasts, rest ECs, late EMT, proliferative fibroblast, rest fibroblasts, late EndMT, *DKK2+* ECs, and activated ECs cells had a strong correlation. This phenomenon prompted us to question why fibroblasts and correlate strongly with ECs (**Figure 1E**). Therefore, we analyzed fibroblasts, and based on a literature search, we analyzed fibroblast subpopulations such as rest fibroblasts, myofibroblasts, and proliferative fibroblasts (**Figures 1F, H**). A comparison of COVID-19 and IPF samples revealed that the, myofibroblast proportion was significantly higher in patients with IPF compared to COVID-19 patients (**Figure 1G**). Interestingly, we also found that myofibroblast expression was much higher in male COVID-19 patients than male patients, but the opposite was true for rest fibroblasts (**Figure 1I**). We also analyzed the expression of entry factors in whole lung cells, and consistent with previous literature reports, *ACE2* was mainly concentrated in respiratory epithelium cells such as AT1 and AT2. However, BSG, CTSL, and FURIN were also abundantly expressed in macrophages and fibroblasts (**Figure 1J**). TMPRSS2 and ACE2 expression were upregulated in COVID-19 patients but BSG, CTSL, FURIN expression was higher in IPF patients, suggesting their role in increased myofibroblast expression in COVID-19 (**Figure 1K**). We also analyzed correlation analysis of entry factors and lung function test (%predicted DLCO) and found that lung function was negatively correlated with the normalized bulk RNA-seq gene expression of the entry factors (*CTSL*, *BSG*, *ACE2*) (**Figure 1L**).

### Single-cell transcriptome sequencing Atlas of EMT stage

**Figure 2:**
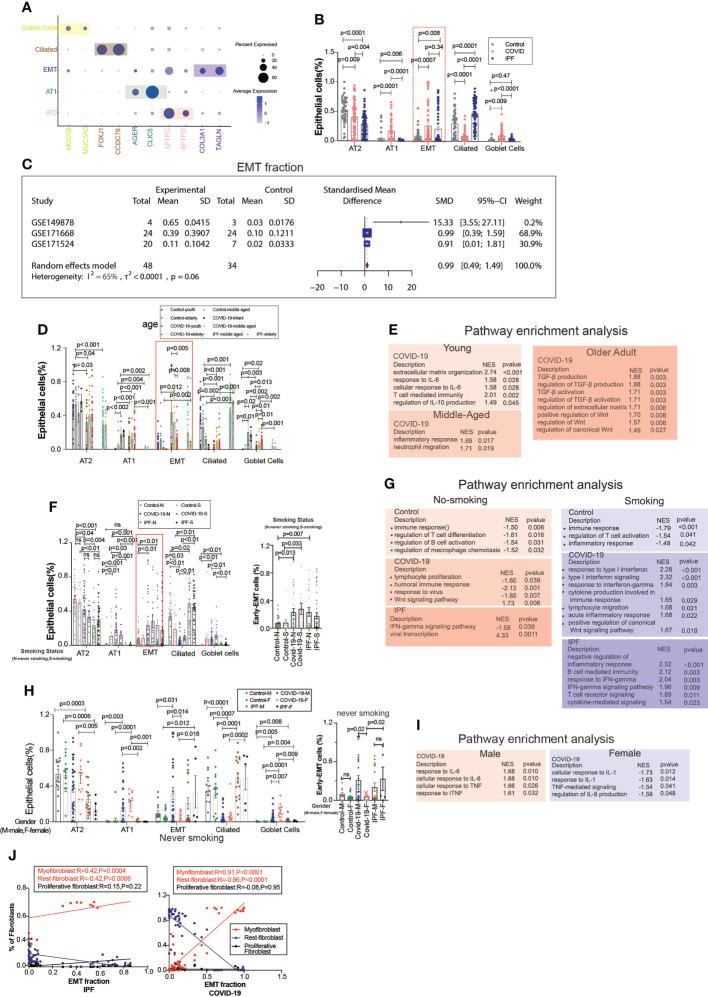
EMT by age, smoking status, and gender. **(A)** Dot chart shows the marker of epithelial subpopulations, including AT1(AGER, CLIC5), AT2(SFTPC, SFTPD), Ciliated (FOXJ1, CCDC78), Goblet cells (MUC5B, MUC5AC), EMT(TAGLN, COL3A1). **(B)** Comparison of the ratios of HC, COVID-19, IPF subpopulations of epithelium. EMT proportion was significantly upregulated in COVID-19 and IPF. **(C)** Forest plot of studies with EMT ratio on the COVID-19 and HC, after excluding a study with only one case and a high heterogeneity study. The analysis included data from 3 studies with a total of 48 COVID-19 and 34 controls. p value for heterogeneity was 0.06, I^2^ was 65%. (SD, Standard deviation). **(D)** Comparison of the ratios of HC, COVID-19, IPF subpopulations of epithelium by age. **(E)** GO (Gene ontology) enrichment analysis was performed on the genes highly expressed in EMT in normal control, COVID-19 and IPF groups in patients of different ages, respectively, and the graphs show the signaling pathways enriched to EMT cell populations in different groups. **(F)** Comparison of the ratios of HC, COVID-19, IPF subpopulations of epithelium by smoking. **(G)** GO enrichment analysis was performed to enrich for genes that were highly expressed in EMT of smoking and non-smoking patients in normal control, COVID-19 and IPF groups, respectively, and the graphs show the signaling pathways enriched in EMT cell populations of different groups. **(H)** Comparison of the ratios of HC, COVID-19, IPF subpopulations of epithelium by sex. **(I)** GO enrichment analysis of the genes highly expressed in EMT in normal control, COVID-19 and IPF groups by sex, respectively. **(J)** Correlation analysis of EMT with myofibroblast, rest fibroblast and proliferative fibroblast in COVID-19 and IPF patients (Pearson test), showing EMT is positively correlated with myofibroblasts.

EMT is a common type of transition, and we divided the epithelium into goblet, ciliated, EMT, AT1, and AT2 cell subgroups, in which the EMT proportion was significantly upregulated in COVID-19 and IPF patients, but there were no significant differences between COVID-19 and IPF (**Figures 2A, B**). After excluding datasets with significant heterogeneity, we performed a meta-analysis using 3 GEO datasets with a pooled SMD of 0.99 and 95% CI (0.49; 1.49) (**Figure 2C**). Surprisingly, the EMT rate was much more significant in the middle-aged group than in the aged group. The middle-aged group was mainly enriched for inflammation-related signaling pathways, and then the aging adult group was enriched for more fibrosis-related signaling pathways (**Figures 2D, E**). Whether classified by smoking or not, we observed that EMT was higher in both COVID-19 patients than in controls, and then IPF was higher in the non-smoking group compared to samples. In COVID-19, smoking is enriched for more interferon-related signaling pathways compared to samples from non-smoking individuals (**Figures 2F, G**). We found that the proportion of EMT was higher in patients who smoked, while in COVID-19 patients, the proportion of EMT was more significant in non-smoking males than in females (**Figures 2H, I**). This phenomenon indicates that males contribute more to EMT progression. EMT is positively correlated with myofibroblasts in COVID-19 patients, and IPF patients also have the same expression trend. Males upregulated inflammation-related signaling pathways, however females were enriched for downregulated inflammatory signaling pathways (**Figure 2J**).

**Figure 3:**
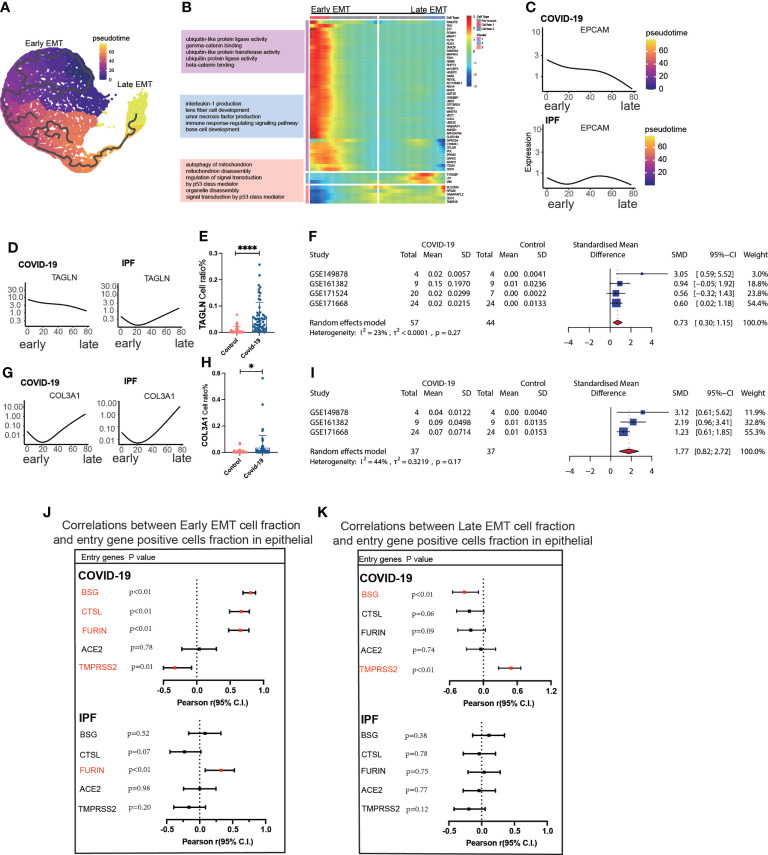
Characteristics of early late-stage EMT. **(A)** Pseudotime projection analysis showing early-stage EMT could evolve to late-stage EMT. **(B)** Single cell proposed time branch point analysis showing genes with progressively lower and higher expression from Early EMT to Late EMT. Each row of the heat map on the right represents a gene, each column is a proposed time point, and the color represents the average expression value of the gene at the current time point, with the color decreasing from red to blue. The left side shows the signaling pathways that the gene set is enriched. **(C)** Proposed time traces of individual genes, showing the change in gene expression from the beginning to the end of the proposed time course for epithelial cell marker genes (EPCAM) in COVID-19 and IPF patients, respectively. **(D)** Time-sensitive trajectories of individual genes, showing the change of Early EMT marker gene (TAGLN) expression from the beginning to the end of the proposed time course in COVID-19 and IPF patients, respectively. **(E)** TAGLN gene positive epithelial cell ratio is higher in COVID-19 than that in HC. **(F)** Forest plot of studies with TAGLN gene positive epithelial cell ratio on the COVID-19 and HC, after excluding a study with only one case. the analysis included data from 4 studies with a total of 57 COVID-19 and 44 controls. p value for heterogeneity was 0.27, I2 was 23%.**(G)** Temporal trajectories of individual genes, showing changes in gene expression of Late EMT marker gene (COL3A1) in COVID-19 and IPF patients by the start to the end of the proposed time course, respectively. **(H)** COL3A1 gene positive epithelial cell ratio is higher in COVID-19 than that in HC. **(I)** Forest plot of studies with COL3A1 gene positive epithelial ratio on the COVID-19 and HC, after excluding a study with only one case and a high heterogeneity study. The analysis included data from 3 studies with a total of 37 COVID-19 and37 controls. p value for heterogeneity was 0.17, I^2^ was 44%. **(J)** Forest plot showing the correlation between Early EMT cell fraction and entry gene positive cells fraction in epithelial cells. And entry factors (BSG, FURIN, CTSL) were positively correlated with the expression of Early EMT in COVID-19 (p < 0.05, Pearson’s r > 0). TMPRSS2 was negatively correlated with the expression of Early EMT in COVID-19 (p < 0.05, Pearson’s r < 0). FURIN was positively correlated with the expression of Early EMT in IPF (p < 0.05, Pearson’s r > 0). **(K)** Forest plot showing the correlation between Late EMT cell fraction and entry gene positive cells fraction in epithelial cells. And entry factor (TMPRSS2) was positively correlated with the expression of Late EMT in COVID-19 (p < 0.05, Pearson’s r > 0). BSG was negatively correlated with the expression of Late EMT in COVID-19 (p < 0.05, Pearson’s r < 0) *P < 0.05,**** P< 0.0001.

Trajectory analysis mentions early and late mesenchymal transitions, and its marker also suggests its transition status (**Figure 3A**). *EPCAM* expression was significantly decreased from early to late stages. TAGLN expression was increased in the early stages but decreased in the late stages. *COL3A1* expression was somewhat lower during the early stages but increased significantly during the late stages (**Figures 3C, D, G**). From early to late stages, Monocel2 displayed the primary enhanced signaling pathways concurrently (**Figure 3B**). *TAGLN* is a typical EMT marker gene that is elevated in COVID-19 patients. A meta-analysis of a dataset of five GEO resulted in a pooled SMD of 0.73, 95 CI% (0.30; 1.15) (**Figures 3D–F**). *COL3A1* is a well-characterized marker of late MT that is increased in COVID-19 patients. The dataset of five GEOs was analyzed using meta-analysis, resulting in a pooled SMD of 1.77, 95 CI% (0.82; 2.72) (**Figures 3G–I**). Further analysis of the correlation between invasion genes showed that the epithelial entry factor genes (*BSG*, *FURIN*, *CTSL*) were positively correlated with the ratio of early EMT in COVID-19, *TMPRSS2* was negatively correlated with the ratio of early EMT in COVID-19, and FURIN was positively correlated with the ratio of early EMT in IPF (**Figure 3J**). *TMPRSS2* was positively correlated with the ratio of late EMT in COVID-19 and *BSG* was negatively correlated with the ratio of late EMT in COVID-19 (**Figure 3K**).

### Single-cell transcriptome sequencing Atlas of EndMT stage

**Figure 4:**
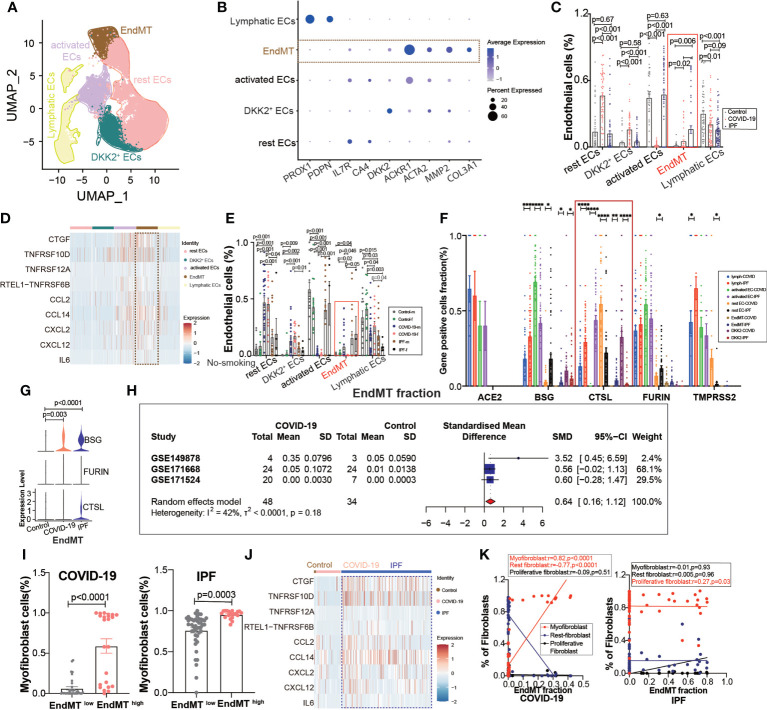
EndMT proportion is upregulated in COVID-19. **(A)** The expression of pulmonary ECs in patients with health control, COVID-19, IPF was demonstrated using UMAP plots with subpopulations marked by a color code. ECs subpopulations including rest ECs, EndMT, *DKK2* ^+^ ECs, activated ECs; Lymphatic ECs. **(B)** Dot chart shows the marker of EndMT, mainly focusing on ACTA2, MMP2, COL3A1; rest ECs (IL7R, CA4), *DKK2* ^+^ ECs (DKK2), activated ECs (ACKR1); Lymphatic ECs(PROX1,PDPN). **(C)** Proportion of ECs among normal control group, COVID-19 group and IPF group, the ratio of EndMT was increased in COVID-19 and IPF than controls. One-way ANOVA was used to compare multiple groups (p<0.05). **(D)** Heatmap shows a comparison of the expression of major cytokines in the cell subpopulations of ECs shows that EndMT is enriched in more cytokines compared to the other groups. **(E)** Comparison of the ratios of HC, COVID-19, IPF subpopulations of ECs by gender in nonsmokers. EndMT ratio in females is lower than that in males with COVID-19. **(F)** Comparison of entry factors (ACE2, BSG, FURIN, CTSL, TMPRSS2) positive cell fraction in ECs subtype of COVID-19 and IPF. **(G)** Comparison of EndMT proportion of entry factors (BSG, FURIN, CTSL) in HC, COVID-19, IPF. **(H)** Forest plot of studies with lung scRNA data on the COVID-19, after excluding a study with only one case and a high heterogeneity study. The analysis included data from 3 studies with a total of 48 COVID-19 and 34 controls. p value for heterogeneity was 0.18, I^2^ was 42%. (SD: Standard deviation). **(I)** Ratio of myofibroblasts in COVID-19 and IPF for EndMT ^low^ versus EndMT ^high^, showing myofibroblast ratio in EndMT ^high^ is higher than that in EndMT ^low^. **(J)** A comparison of the expression of major cytokines in the EndMT shows that COVID-19 and IPF are enriched in more profibrotic cytokines compared to HC group. **(K)** Correlation analysis of EndMT with myofibroblast, rest fibroblast and profilerative fibroblast in COVID-19 and IPF patients (Pearson test), showing EndMT is positively correlated with myofibroblasts in COVID-19 patients. *P < 0.05, **P < 0.01, ***P < 0.001,****P < 0.0001.

A further exploration of the potential cause of elevated fibroblast expression in COVID-19, revealed subpopulations of endothelium cells based on the high correlation between fibroblasts and endothelial cells (**Figure 1D**), and we found a subpopulation of endothelial cells with EndMT as in IPF (**Figure 4A**), which highly expresses the marker gene for subpopulations (**Figure 4B**). We divided the subgroups of endothelial cells into lymphatic ECs, EndMT, *DKK2+* ECs, activated ECs, and rest ECs. Comparing the endothelium subgroup ratios in ECs, we found that the ratio of EndMT was increased in COVID-19 and higher in IPF than in the controls (**Figure 4C**). We showed common inflammation-associated cytokines through different subpopulations, implying an abundant expression of EndMT(**Figure 4D**). To exclude the effect of smoking, we selected nonsmoking patients for comparison, and the results suggested that the proportion of EndMT was also higher in males than in females in COVID-19 patients (**Figure 4E**). We compared the proportion of entry factor genes in Lymphatic ECs, EndMT, *DKK2*+ ECs, activated ECs, and rest ECs in COVID-19 and IPF (**Figures 4F, G**). The findings suggest that EndMT plays a unique function in COVID-19, and meta-analysis of data from several GEO databases indicates that the EndMT proportion in COVID-19 vs. control pooled SMD was 0.64, 95% CI [0.16; 1.12] (**Figure 4H**). After dividing the data into two groups, EndMT^low^ and EndMT^high^, the latter group was found to have a higher proportion of myofibroblasts (**Figure 4I**). A hypothesis was then raised about whether EndMT led to the increase in fibroblasts. Enrichment analysis of cytokine expression in the EndMT subpopulation revealed high expression of cytokines associated with fibrosis in COVID-19 and IPF (**Figure 4J**). The EndMT ratio was positively correlated with myofibroblast cells and negatively correlated with rest fibroblast (**Figure 4K**).

**Figure 5:**
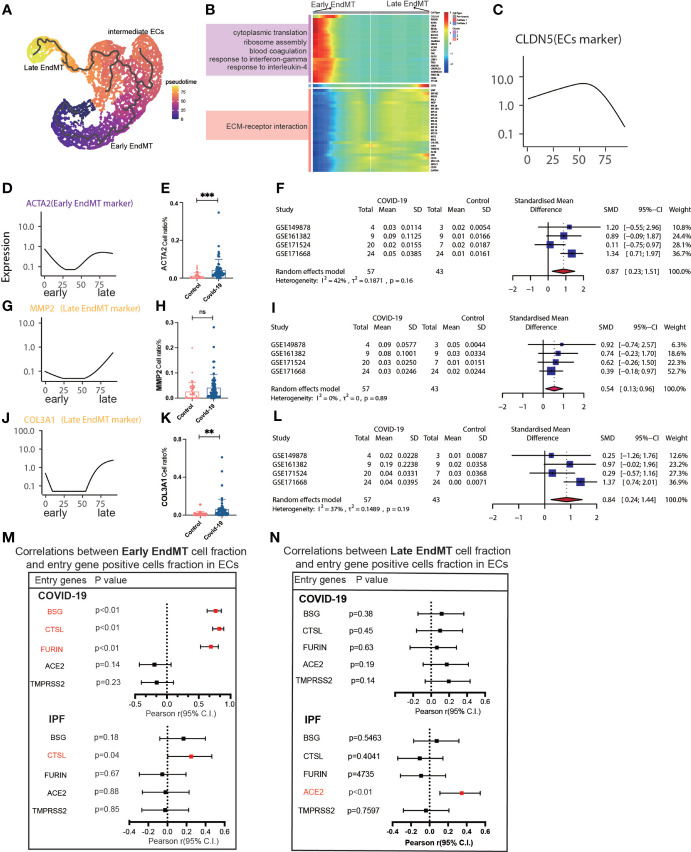
Characteristics of early and late stage EndMT. **(A)** Pseudotime projection analysis showing early-stage EndMT could evolve to late-stage EndMT. **(B)** Single cell proposed time branch point analysis showing genes with progressively lower and higher expression from Early EndMT to Late EndMT. Each row of the heat map on the right represents a gene, each column is a proposed time point, and the color represents the average expression value of the gene at the current time point, with the color decreasing from red to blue. The left side shows the signaling pathways that the gene set is enriched to. **(C)** The proposed time trajectory of a single gene, showing the change in gene expression of the ECs marker gene (CLDN5) from the beginning to the end of the proposed time course. **(D)** Trajectories of individual genes, showing changes in gene expression of Early EndMT marker genes (ACTA2) from the beginning to the end of the proposed time course. **(E)** ACTA2 gene positive ECs ratio is higher in COVID-19 than that in HC. **(F)** Forest plot of studies with ACTA2 gene positive ECs ratio on the COVID-19 and HC, after excluding a study with only one case. The analysis included data from 4 studies with a total of 57 COVID-19 and 43 controls. p value for heterogeneity was 0.16, I2 was 42%. **(G)** Temporal trajectories of individual genes showing changes in gene expression of Late EndMT marker gene (MMP2) from the beginning to the end of the proposed time course. **(H)** MMP2 gene positive ECs ratio is higher in COVID-19 than that in HC. **(I)** Forest plot of studies with MMP2 gene positive ECs ratio on the COVID-19 and HC, after excluding a study with only one case. The analysis included data from 4 studies with a total of 57 COVID-19 and 43 controls. p value for heterogeneity was 0.89, I2 was 0%. **(J)** Trajectories of individual genes showing changes in gene expression of the Late EndMT marker gene (COL3A1) from the beginning to the end of the proposed time course. **(K)** COL3A1 gene positive ECs ratio is higher in COVID-19 than that in HC. **(L)** Forest plot of studies with COL3A1 gene positive ECs ratio on the COVID-19 and HC, after excluding a study with only one case. The analysis included data from 4 studies with a total of 57 COVID-19 and 43 controls. p value for heterogeneity was 0.19, I^2^ was 37%. **(M)** Forest plot showing the correlation between Early EndMT cell fraction and entry gene positive cells fraction in ECs. Entry factors (BSG, FURIN, CTSL) was positively correlated with the expression of Early EndMT in COVID-19 (p < 0.05, Pearson’s r > 0). CTSL was positively correlated with the expression of Early EndMT in IPF (p < 0.05, Pearson’s r > 0). **(N)** Forest plot showing the correlation between Late EndMT cell fraction and entry gene positive cells fraction in ECs. Entry factors (ACE2) was positively correlated with the expression of Late EndMT in COVID-19 (p < 0.05, Pearson’s r > 0). **P < 0.01, ***P < 0.001; NS, P > 0.05.

We then tried to determine whether EndMT has a series of genes that can affect COVID-19 and IPF MT by trajectory analysis and classified EndMT into early, intermediate, and late stages (**Figure 5A**). The enrichment of genes in early and late stages demonstrates that the interferon-gamma signaling pathway is upregulated in early stages. Simultaneously, late stages exhibit increased expression of the ECM-receptor interaction signaling pathway (**Figure 5B**). The expression of marker genes associated with EndMT also suggested early, intermediate, and late stages (**Figure 5C**). *ACTA2* expression was low in the intermediate stage but rapidly increased in the late stage (**Figure 5D**). Calculating the fraction of *ACTA2* present throughout the early stages of the disease reveals that the expression in COIVD-19 is more significant than in controls (**Figure 5E**). By screening the marker genes of early stage EndMT, the marker gene at the early stage was finally identified as *ACTA2* with an SMD of 0.87,95% CI (0.23,1.51) for COVID-19 vs. control in 5 GEO datasets following meta-analysis (**Figure 5F**). Subsequently, MMP2 and COL3A1 were used to identify the late-stage marker gene (**Figures 5G, J**). COL3A1 expression was increased in COVID-19 compared to controls (**Figure 5K**), and although all GSE statistics of MMP2-positive cells were not significant (**Figure 5H**), MMP2-positive cells were statistically differentially upregulated in late stage of EndMT by meta-analysis of GSE data. Using meta-analysis, we demonstrated that the MMP2 pooled SMD in 5 distinct GEO datasets was 0.54, 95% CI (0.13.0.96) (**Figure 5I**), and ultimately, meta-analysis revealed that the COL3A1 pooled SMD of late stages of COVID-19 was 0.84, 95% CI (0.24,1.44) when compared to controls (**Figure 5L**). Further analysis of the correlation with invasion genes showed that the entry factor genes (*BSG, FURIN, and CTSL*) were positively correlated with the ratio of early EndMT in COVID-19, and *CTSL* was positively correlated with the ratio of early EndMT in IPF (**Figure 5M**). *ACE2* was positively correlated with the ratio of late EndMT in IPF (**Figure 5N**).

## Discussion

To our knowledge, this first single-cell meta-analysis that describes MT-related clusters in COVID-19 patients. When we examined the MT transition state in COVID-19 using IPF with MT and a high number of fibroblasts as a control, we identified a substantial link between fibroblasts and MT across all subpopulations. By analyzing the correlation of each subgroup, we screened the relevant subgroups of MT (i.e., EndMT and EMT). COVID-19 patients exhibited a increased MT than healthy controls, but less than IPF patients. We further found that male patients had a higher proportion of cells in EMT and EndMT. Several studies have shown a higher prevalence of pulmonary fibrosis after COVID-19 in males. This is likely because males are more exposed to fibrotic triggers, such as occupational agents (26–28).

In EMT, young people are more enriched in inflammation such as *IL-6* signaling pathways, aging adults are enriched with more fibrosis-promoting signaling pathways such as TGF-b. Increased levels of TGF-b, an anti-inflammatory but profibrotic cytokine, might be the leading cause of EMT in aging adults (29). Smoking COVID-19 patients showed a higher percentage of EMT, but were more enriched in the interferon signaling pathway, compared to non-smoking COVID-19 patients. Despite early reports to the opposite, there is mounting evidence that individuals with severe COVID-19 have a strong type I interferon response, as opposed to the delayed, potentially suppressed response observed early in infection (30). Through various pathways, a potent type I interferon response might increase hyperinflammation in the progression to severe COVID-19 (31). Insights into the therapeutic use of type I interferon in patients with MT will come from an improved knowledge of the functions of type I interferon at various stages of the disease and in patients who are non-smokers vs. smokers.

MT is involved in pulmonary fibrosis and vascular remodeling in the pathogenesis of IPF. Several triggers and pathways are associated with the EMT and EndMT (32). Archana et al. have shown that EndMT markers(N-cadherin, S100A4, and vimentin) are increased in the arterial layers (intima, media, and adventitia) of IPF patients (33). Similarly, during the EndMT process, active ECs express adhesion molecules, such as intercellular adhesion molecule-1 (*ICAM-1*) and vascular cell adhesion molecule-1 (*VCAM-1*), which enhance EndMT formation in our results. Moreover, ICAM-1 is also an adhesion molecule for another virus, such as human rhinovirus (34), influenza virus (35), and HIV (36). It is reasonable to speculate that with the activation of ICAM-1, the risk of associated secondary infection may also increase.

The mesenchymal cells we identified are divided into early and late stage. Compared to IPF, these cells are related to the expression of entry factors such as *BSG*, *CTSL*, and *FURIN*. Sohal et al. indicate that ILDs, especially IPF, have a higher risk of developing severe COVID-19 infection and post-COVID-19 interstitial pulmonary fibrosis. They found TGF-β1 and α-smooth muscle actin (myofibroblast marker) are in similar areas as COVID-19 markers. They suggest that myofibroblasts and surrounding tissue secrete growth factors which could further affect COVID-19 adhesion proteins/cofactors and post-COVID-19 interstitial pulmonary fibrosis (37). Our study confirms that entry factors are negatively correlated with early EndMT cell fraction and are positively correlated with fibroblasts. This suggests that the conversion of activated ECs to EndMT is linked to mesenchymal conversion of ECs in the early phases. Therefore, this provides a strategy for the early prevention of pulmonary fibrosis in COVID-19 patients.

Improvements in patient stratification and therapeutic approaches for lung fibrosis will be contingent on the capacity to reprogram EMT (38). EMT has been hypothesized as a source of myofibroblast also in SSc (39) and EMT-related pathways (40). Early EMT leads to activation fibrosis as a result of the downregulation and/or destruction of junctional components like *TAGLN*. The late EMT mesenchymal marker *COL3A1* increases along with the slow loss of *EPCAM*, a marker of epithelial cells (41). Late EMT has been classically associated with fibrosis in organs such as the kidneys, liver, and lungs. It is interesting to note that, in consistent with our data, the EMT status of primary tumors was not consistently associated with poor patient survival (38). Our study suggests that late EMT and invasive genes are almost independent, and early EMT is associated with invasive genes. The process of EndMT can also represent a therapeutic target in the early stages of the disease (42). Early, immature stress fibers are present in the early stages of EndMT. Late stages of EndMT, however, may be identified by matured stress fibers formed by microfilaments (43). The inflammatory leukocytes engaged in the early stages of the illness trigger the EndMT, which has also been discovered in lung fibrosis (44). Therefore, late EndMT could contribute to aggravating the pro-fibrotic signaling in lung fibrosis. Late EndMT, then, could contribute to exacerbating the pro-fibrotic TGF-β signaling in lung fibrosis. According to this principle, we chose *ACTA2* as the early marker and also MMP2 and *COL3A1* as the late markers. *ACTA2* was only transiently displayed in the early EndMT, but *MMP2* and *COL3A1* were subsequently upregulated through further remodeling (45).

It is important to note that our study has some limitations:1. First, we not count whole organ MT because this included all the up-regulation of the entry factor. This data will be included in our subsequent studies. Second, no in vitro or in vivo animal experiments were performed to validate the key genes. Third, we did not validate COVID-19 patient samples. Fourth, no SARS-CoV-2 positive cells were extracted for further analysis. Finally, the correlation between MT and entry factor was inadequate and required further exploration.

## Conclusion

In conclusion, for the first time, we have analyzed ECs as well as epithelial MT subpopulations in COVID-19 patients, establishing the correlation between their MT subpopulations with irreversible myofibroblasts. However, although entry factors are also highly expressed in IFP, it is not associated with the MT of IPF. Thus, we further explored the mesenchymal-associated cluster's genes and found that some genes are expressed both in COVID-19 patients and IPF patients. This suggests potent targets that could reverse MT in COVID-19 and IPF.

## Data availability statement

The original contributions presented in the study are included in the article/**Supplementary Material**. Further inquiries can be directed to the corresponding authors.

## Author contributions

LZ, XZ, and DL conceived the topic for this study. LZ, XZ, MZ, and XT performed single-cell RNA sequencing. LZ, XZ, XW, SL, RY, MZ, YX, XT, CT, and DL contributed to the writing and revising of the manuscript. DL, and CT critically revised the manuscript. All authors listed have read and approved the manuscript before submission.

## Funding

Postdoctoral Funding of the Personnel Office-Special Fund for Postdoctoral New Crown Epidemic Prevention and Control (0040204153344), National Science Foundation for Young Scientists of China (81900065), Project supported by the Natural Science Foundation of Sichuan province (2022NSFSC1394), the Youth Innovation Project of Sichuan Medical Association (Q21018), and Chengdu Medical Research Projects (2022516).

## Acknowledgments

The authors thank Dr. Jianming Zeng (University of Macau), and all the members of his bioinformatics team, biotrainee, for generously sharing their experience and codes.

## Conflict of interest

Author MZ is employed by the Oncology Bussiness Department, Novogene Co., Ltd, Beijing, China. 

The remaining authors declare that the research was conducted in the absence of any commercial or financial relationships that could be construed as a potential conflict of interest.

## Publisher’s note

All claims expressed in this article are solely those of the authors and do not necessarily represent those of their affiliated organizations, or those of the publisher, the editors and the reviewers. Any product that may be evaluated in this article, or claim that may be made by its manufacturer, is not guaranteed or endorsed by the publisher.

## Abbreviations

COVID-19, Coronavirus disease 2019; MT, Mesenchymal transition; EndMT, Endothelial-mesenchymal transition; EMT, Epithelial-mesenchymal transition; IPF, Idiopathic pulmonary fibrosis.
