# Supplementary material for: Single cell meta-analysis of EndMT and EMT state in COVID-19
Source: Front Immunol. 2022 Sep 29;13:976512. doi: 10.3389/fimmu.2022.976512 (PMC9558222; doi:10.3389/fimmu.2022.976512)
Supplement: Supplementary file 1 [file DataSheet_1.pdf]

sFigure1: Quality control (QC) of each dataset

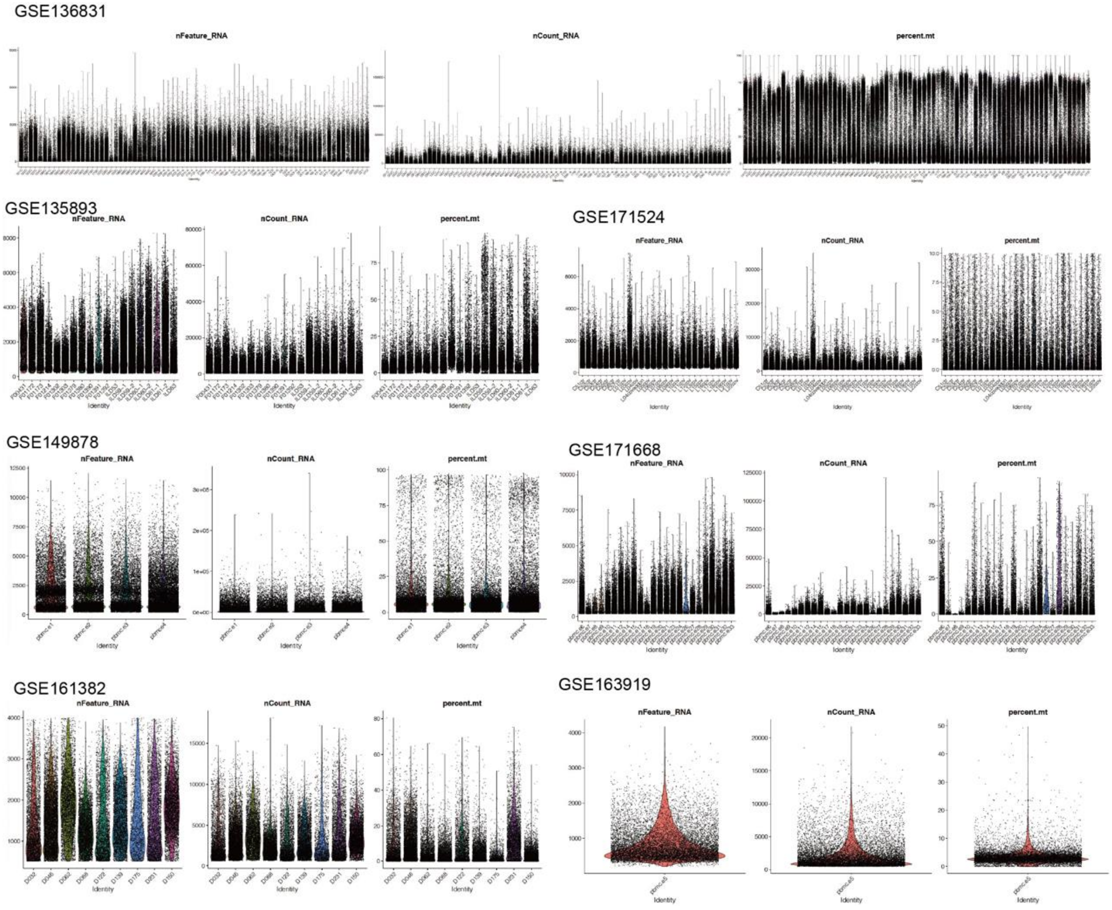

sFigure1: Quality control (QC) of each dataset. Scatterplot illustrating the number of genes, RNA molecules (UMIs) and the percentage of mitochondrial genes in each cell of the lung samples.

sFigure2: Batch effect correction

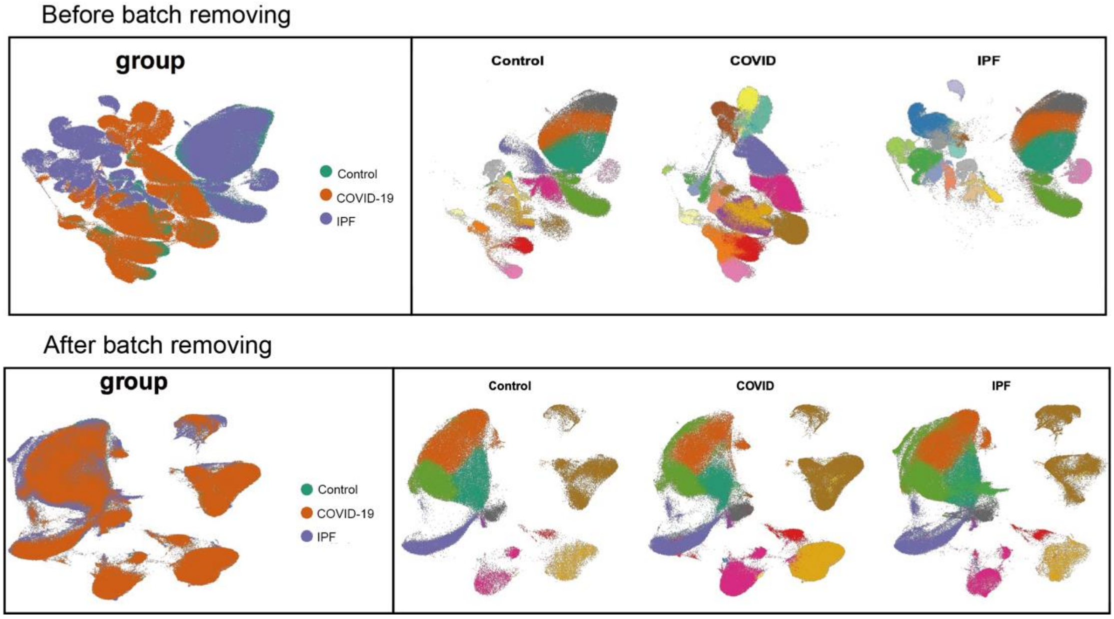

sFigure2: UMAP visualization before and after batch correction. The separation of batches is clearly visible before batch correction and less visible afterwards. Batch correction was performed using Harmony.

sFigure3: The markers of early and late MT

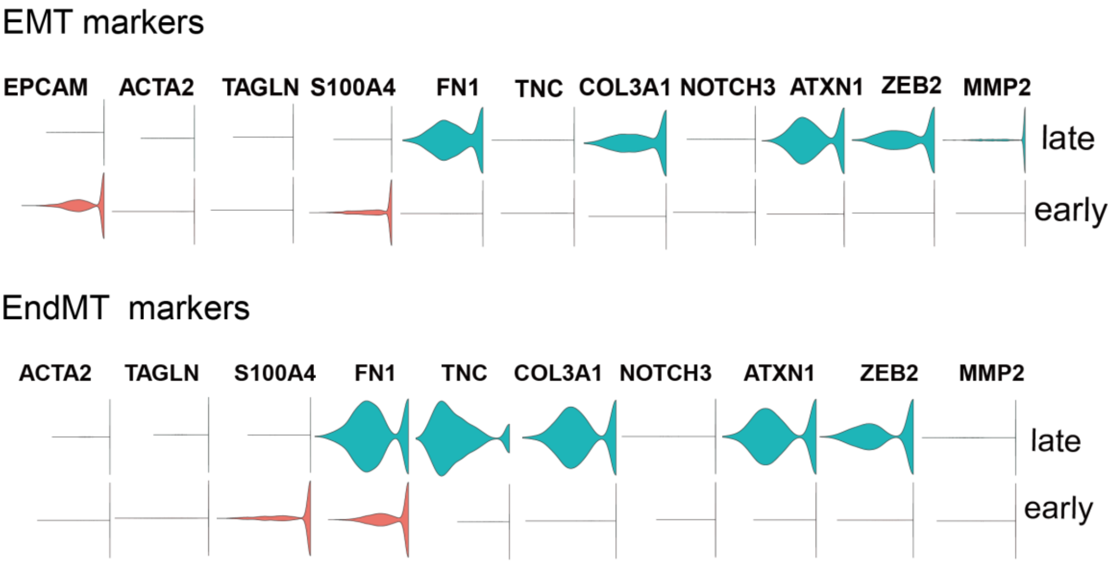

sFigure3: Violin diagram shows the marker genes of early and late MT. early-stage MT mainly focusing on ACTA2, TAGLN,S100A4.late-stage MT mainly focusing on FN1, TNC, COL3A1, NOTCH3, ATXN1, ZEB2, MMP2.

sFigure4: Expression of each cell subset marker gene in all cell subsets

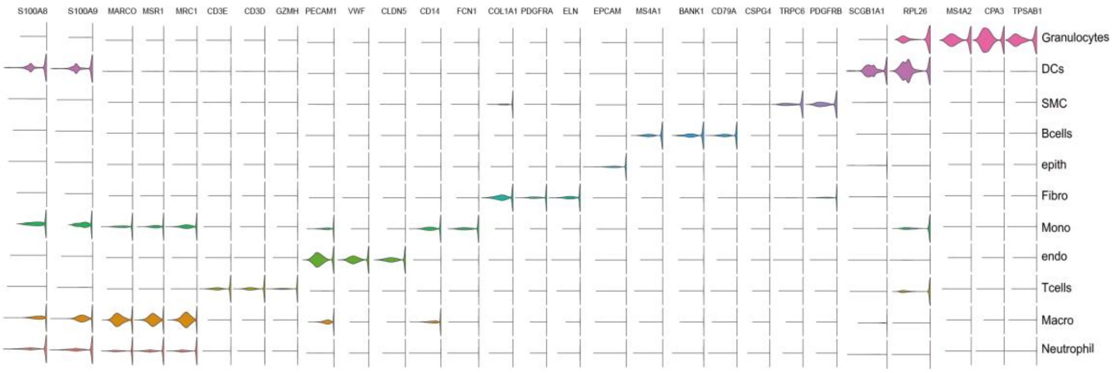

sFigure4: Violin diagram shows expression of each cell subset marker gene in all cell subsets: Endothelial cells (endo)：PECAM1, VWF, CLDN5；Macrophages(Macro)：MARCO, MSR1, MRC1；Tcells：CD3E, CD3D, GZMH；Granulocytes：MS4A2, CPA3, TPSAB1；Bcells：MS4A1, BANK1, CD79A；Monocytes(Mono)：CD14, FCN1；Neutrophil：S100A8, S100A9；Epithelial cells(epith)：EPCAM；Fibroblast(Fibro)：COL1A1, PDGFRA, ELN；SMC：SCGB1A1，RPL26.
